# Implementation of an optimal fluid management protocol using the PiCCO system delays development of ARDS secondary to severe sepsis

**DOI:** 10.1186/cc10850

**Published:** 2012-03-20

**Authors:** N Saito, T Yagi, Y Hara, H Matsumoto, K Mashiko

**Affiliations:** 1 Chiba-Hokusoh Hospital, Nippon Medical School, Chiba, Japan

## Introduction

Acute respiratory distress syndrome (ARDS) is often associated with sepsis, and one recommended approach in the fluid management of ARDS is to keep sepses dry. On the other hand, optimal fluid management after the early phase of septic shock remains unknown. An excessive positive fluid balance in patients with septic shock is associated with increased mortality and morbidity. We implemented an optimal fluid management (OFM) protocol using the PiCCO system from April 2009. The purpose of this study was to examine the effect of the OFM protocol in comparison with historical controls.

## Methods

A retrospective study was conducted in a Japanese mixed ICU of a tertiary-care teaching hospital from July 2007 to March 2011. Our protocol includes daily volume assessment using the PiCCO system after ICU admission and a change of fluid therapy after evaluation; for example, additional diuretic use or fluid restriction. We retrospectively analyzed 96 consecutive patients with severe sepsis or septic shock who required mechanical ventilation between July 2007 and December 2010. We divided patients into the OFM protocol group (P; *n *= 49; April 2009 to December 2010) and the control group (C; *n *= 47; July 2007 to March 2009) and compared their clinical and laboratory data.

## Results

Median (IQR) age was 69.5 (55.5 to 78.5) years, and the median APACHE II score and SOFA score were 23.0 (19.0 to 27.0) and 10.0 (7.0 to 12.0), respectively. The proportion of patients with septic shock was 75%. There was no difference in patient characteristics between the two groups. At 28 days, the mortality rate was similar in both groups (P: 14.3%; C: 17.0%; *P *= 0.78). The incidence of ARDS after ICU admission in the P group was significantly lower than that in the C group (P: 20.4%; C: 57.4%; *P *= 0.02). In addition, the onset of ARDS in the P group occurred later than that in the C group (*P *< 0.01). Achievement of a negative water balance in the P group occurred earlier than in the C group. The incidence of AKI (RIFLE criteria: failure) and another organ failure was similar in both groups. Multivariate regression analysis revealed that the OFM protocol independently suppressed the onset of ARDS (OR 0.17 (*P *= 0.001; 95% CI: 0.06 to 0.51)).

## Conclusion

Implementation of an OFM protocol using the PiCCO system significantly decreased the development of ARDS secondary to severe sepsis with no other complications.

